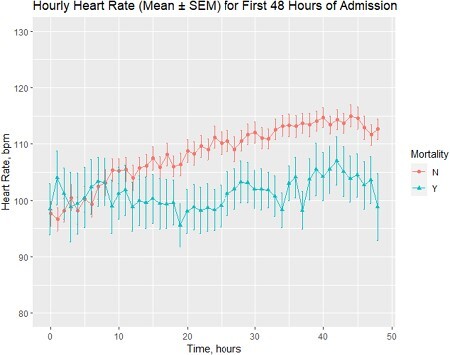# 700 Early Heart Rate Trend Predicts Burn Mortality

**DOI:** 10.1093/jbcr/irae036.245

**Published:** 2024-04-17

**Authors:** Arushi Biswas, Keith T Kuo, Shanmuga Priya Rajagopalan, Terrence Tsou, Tomer Lagziel, Madhu Subramanian, Carisa M M Cooney, Julie A Caffrey

**Affiliations:** Johns Hopkins University School of Medicine, Baltimore, MD; Johns Hopkins Department of Plastic and Reconstructive Surgery, Baltimore, MD; Johns Hopkins School of Medicine, Baltimore, MD; Department of Plastic Surgery, Johns Hopkins School of Medicine, Baltimore, MD; Johns Hopkins University School of Medicine, Baltimore, MD; Johns Hopkins Department of Plastic and Reconstructive Surgery, Baltimore, MD; Johns Hopkins School of Medicine, Baltimore, MD; Department of Plastic Surgery, Johns Hopkins School of Medicine, Baltimore, MD; Johns Hopkins University School of Medicine, Baltimore, MD; Johns Hopkins Department of Plastic and Reconstructive Surgery, Baltimore, MD; Johns Hopkins School of Medicine, Baltimore, MD; Department of Plastic Surgery, Johns Hopkins School of Medicine, Baltimore, MD; Johns Hopkins University School of Medicine, Baltimore, MD; Johns Hopkins Department of Plastic and Reconstructive Surgery, Baltimore, MD; Johns Hopkins School of Medicine, Baltimore, MD; Department of Plastic Surgery, Johns Hopkins School of Medicine, Baltimore, MD; Johns Hopkins University School of Medicine, Baltimore, MD; Johns Hopkins Department of Plastic and Reconstructive Surgery, Baltimore, MD; Johns Hopkins School of Medicine, Baltimore, MD; Department of Plastic Surgery, Johns Hopkins School of Medicine, Baltimore, MD; Johns Hopkins University School of Medicine, Baltimore, MD; Johns Hopkins Department of Plastic and Reconstructive Surgery, Baltimore, MD; Johns Hopkins School of Medicine, Baltimore, MD; Department of Plastic Surgery, Johns Hopkins School of Medicine, Baltimore, MD; Johns Hopkins University School of Medicine, Baltimore, MD; Johns Hopkins Department of Plastic and Reconstructive Surgery, Baltimore, MD; Johns Hopkins School of Medicine, Baltimore, MD; Department of Plastic Surgery, Johns Hopkins School of Medicine, Baltimore, MD; Johns Hopkins University School of Medicine, Baltimore, MD; Johns Hopkins Department of Plastic and Reconstructive Surgery, Baltimore, MD; Johns Hopkins School of Medicine, Baltimore, MD; Department of Plastic Surgery, Johns Hopkins School of Medicine, Baltimore, MD

## Abstract

**Introduction:**

During post-burn hypermetabolism, tachycardia is considered a compensatory response to sustain cardiac output and oxygenation to essential organs. Although heart rate variability predicts mortality in burn patients, many centers lack access to non-invasive measurement methods. We hypothesized that heart rate trend in the first 48 hours of admission (measured via vital signs monitor) could predict mortality in burn patients.

**Methods:**

We performed a retrospective review of 128 patients treated for burns with TBSA ≥ 20% at a single institution between 2016 and 2021. We extracted demographics, burn characteristics (e.g. location, mechanism, depth, presence of inhalation injury), complications (surgical and hospital-acquired), and hourly vital signs for the first 48 hours of admission. We performed bivariate analyses to determine associations between mortality within 90 days of discharge and patient and burn characteristics. We conducted multivariate regression to examine the associations between heart rate trends and 90-day post-discharge mortality, adjusting for confounding variables.

**Results:**

Of 128 patients, 73% were male. Median age at time of burn was 52 (IQR, 38-66). Mean TBSA was 40%. The most common burn mechanism was flame (86%). 29 (23%) patients died within 90 days of discharge. Bivariate analysis demonstrated significant associations between mortality and age, TBSA, length of hospital stay, smoking status, whether patients had surgical intervention (e.g. excision, debridement), and number of follow-up visits. On average, patients who survived experienced a 20% increase in heart rate between the 1st and 48th hour of admission, compared to a 9% increase in patients who died. Multivariate regression, controlling for age, TBSA, inhalation injury, renal injury, and vasopressor usage on admission, demonstrated that heart rate trend in the first 48 hours of admission was a significant predictor of mortality within 90 days of discharge (p< 0.001).

**Conclusions:**

Burn patients whose heart rate increased less than 20% in the first 48 hours of admission exhibit a higher mortality risk. This underscores the need for intensified clinical surveillance and intervention for such high-risk patients, optimizing outcomes.

**Applicability of Research to Practice:**

The conclusion drawn from this study emphasizes the role of initial heart rate trends in recognizing burn patients at elevated mortality risk, serving as a guideline for clinicians in allocating attention and medical resources more efficiently.